# Supplementary material for: Macrophages migrate persistently and directionally upon entering 2D confinement in the presence of extracellular matrix
Source: bioRxiv. 2025 May 15:2025.05.15.654321. Preprint. [Version 1] doi: 10.1101/2025.05.15.654321 (PMC12247838; doi:10.1101/2025.05.15.654321)
Supplement: 1 — Supplemental Figure 1. Additional quantification related to Figure 1, part 1. (A) Left: Confocal image of Lifeact-Scarlet expressing cells (red staining) within an agarose gel containing fluorescent dextran (white staining). Side projections corresponding to the vertical and horizontal lines across this image are at the right and bottom of this image, respectively. Right: The vertical side projection has been rotated (top) and color-coded markers have been included to mark the bottom (Z1), middle (Z2) and top (Z3) of the image series consisting of 43 total images, which is composed of z slices taken at 0.3-micron intervals. Of these, 17 slices span the width of the indicated cell. The images corresponding to the three representative positions (Z1, Z2, Z3) are taken from the 43 total image slices and are reproduced with color-coded borders. As with the image on the left, dextran is colored white and Lifeact-Scarlet is colored red. Scale Bar = 20 microns. (B) Example images of LA-Scarlet staining in live macrophages in unconfined (top) or agarose-confined (bottom) settings. Scale bar = 20 microns and both images are at the same scale. Insets have been maintained at the same scale relative to each other. Supplemental Figure 2. Additional quantification related to Figure 1, part 2. (A) Persistence (left) and Euclidean distance (right) of Far +/− and Close +/− macrophages migrating under agarose with media only. Both values were calculated from the same dataset as Fig. 1C. Experimental means and SEM are represented with black symbols and all cell migration tracks are plotted and each experimental run is color- and shape-coded (circle, square, or triangle). Statistical analysis was assessed with Kruskal–Wallis and Dunn multiple comparisons test. Ns= not significant. Far- n = 123 tracks, Far+ n = 104 tracks, Close+ n = 139 tracks, Close- n = 123 tracks. These data were pooled from 3 independent experiments. ns = not significant (B) Persistence (left) and Euclidean distance [file NIHPP2025.05.15.654321V1-supplement-1.pdf]

bioRxiv preprint doi: https://doi.org/10.1101/2025.05.15.654321; this version posted May 15, 2025. The copyright holder for this preprint (which was not certified by peer review) is the author/funder. This article is a US Government work. It is not subject to copyright under 17 USC 105 and is also made available for use under a CC0 license.

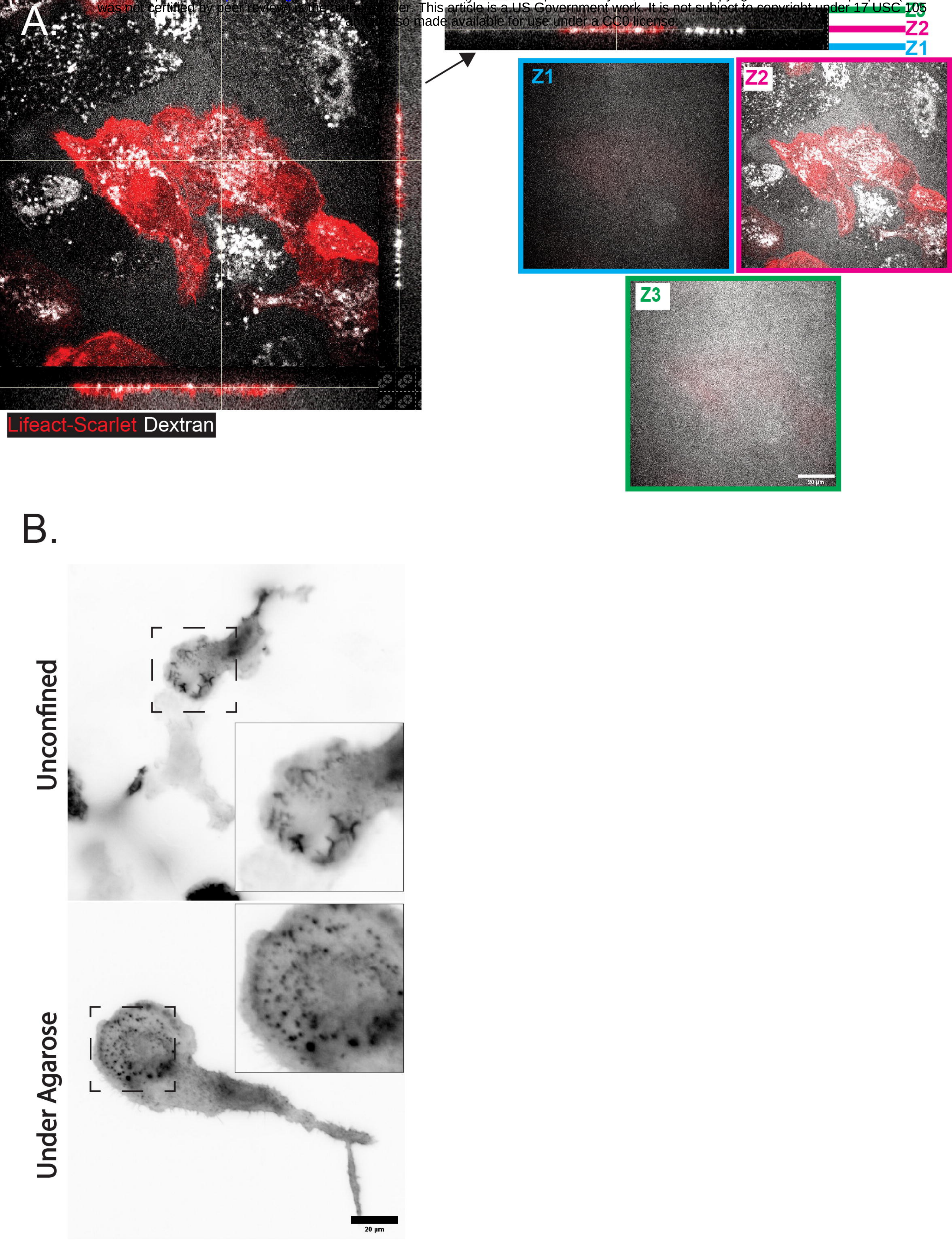

Stinson et al., Supplemental Figure 1

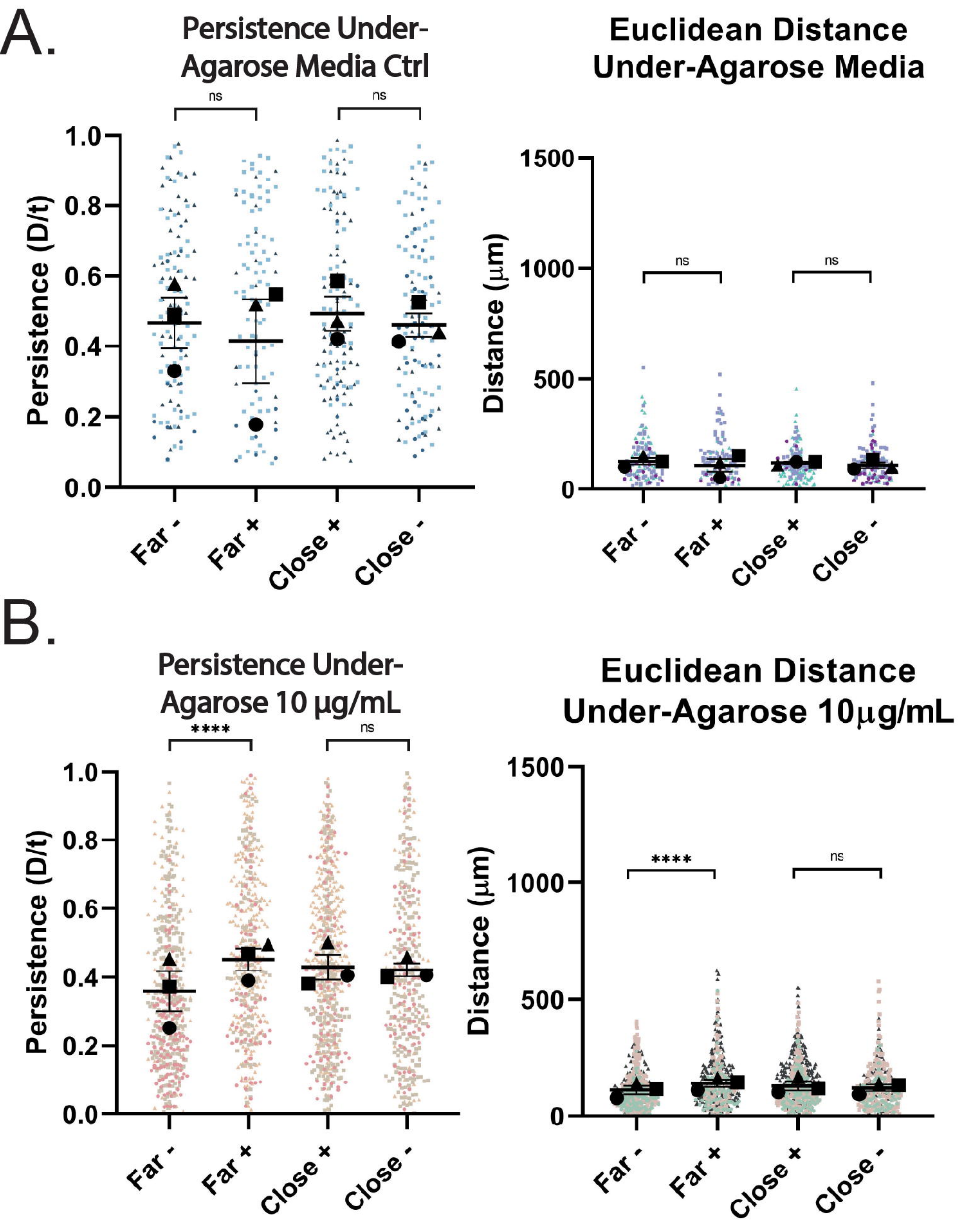

Stinson et al., Supplemental Figure 2

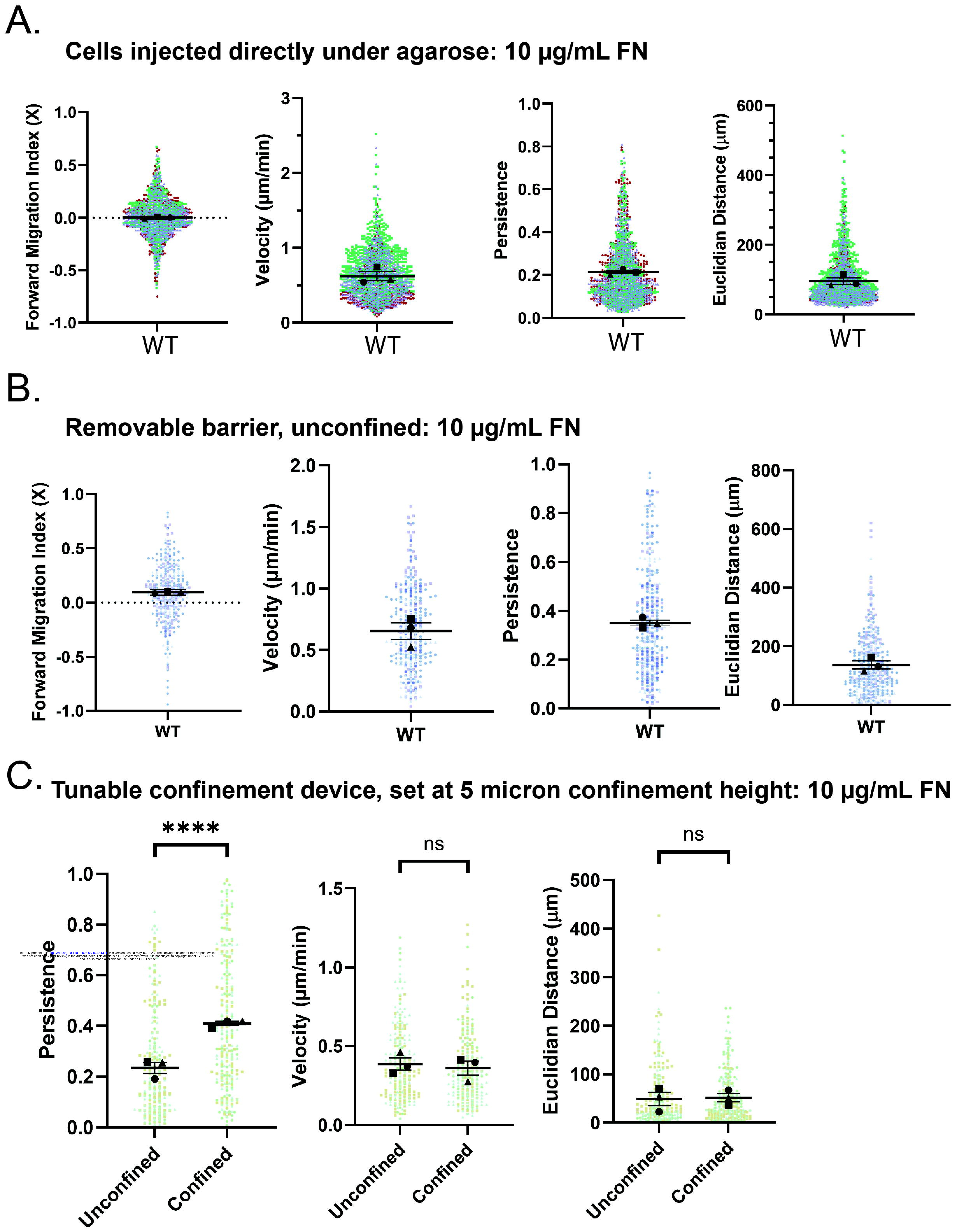

bioRxiv preprint doi: https://doi.org/10.1101/2025.05.15.654321; this version posted May 15, 2025. The copyright holder for this preprint (which was not certified by peer review) is the author/funder. This article is a US Government work. It is not subject to copyright under 17 USC 105 and is also made available for use under a CC0 license.

Stinson et al., Supplemental Figure 3

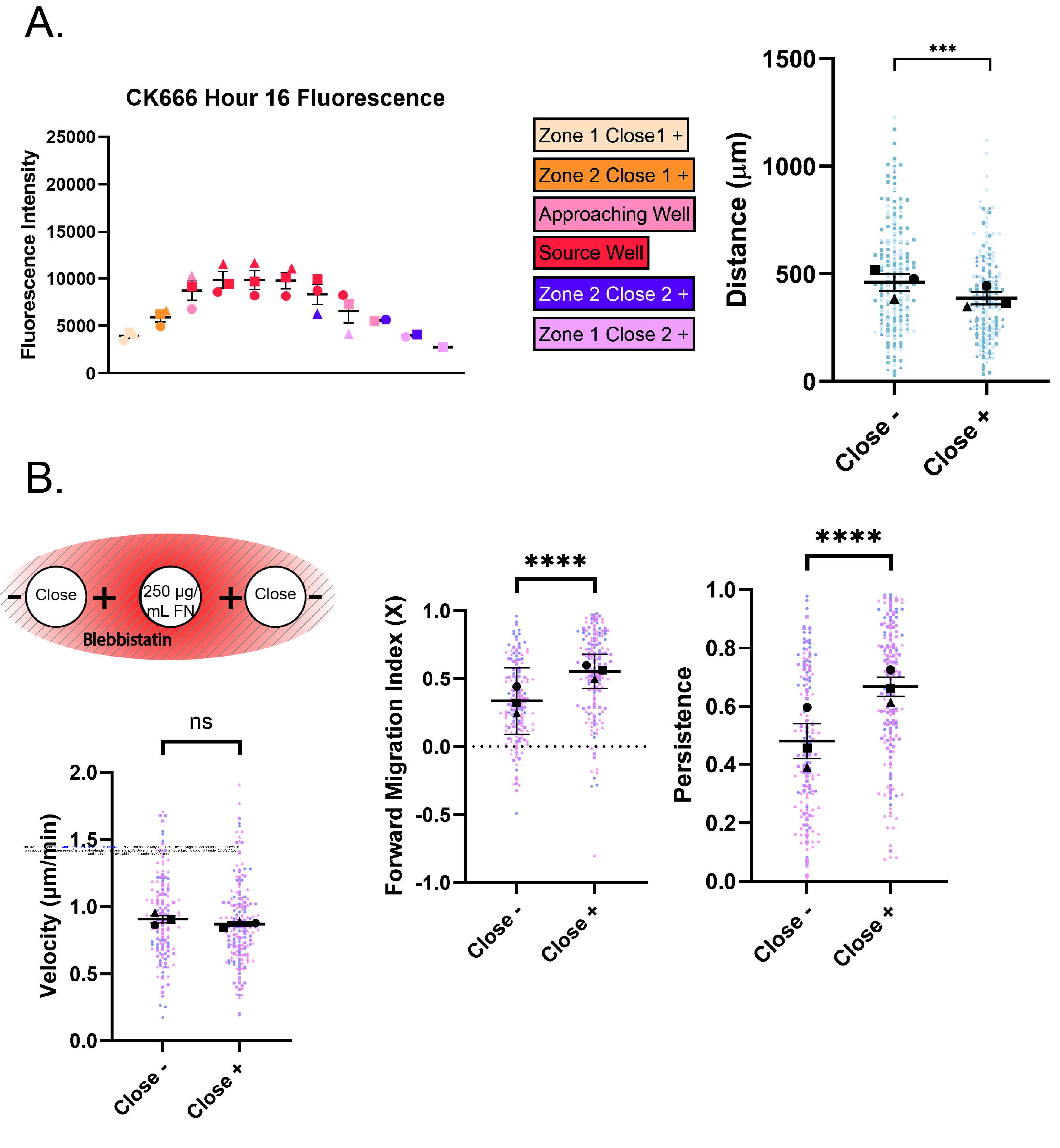

bioRxiv preprint doi: https://doi.org/10.1101/2025.05.15.654321; this version posted May 15, 2025. The copyright holder for this preprint (which was not certified by peer review) is the author/funder. This article is a US Government work. It is not subject to copyright under 17 USC 105 and is also made available for use under a CC0 license.

Stinson et al., Supplemental Figure 4

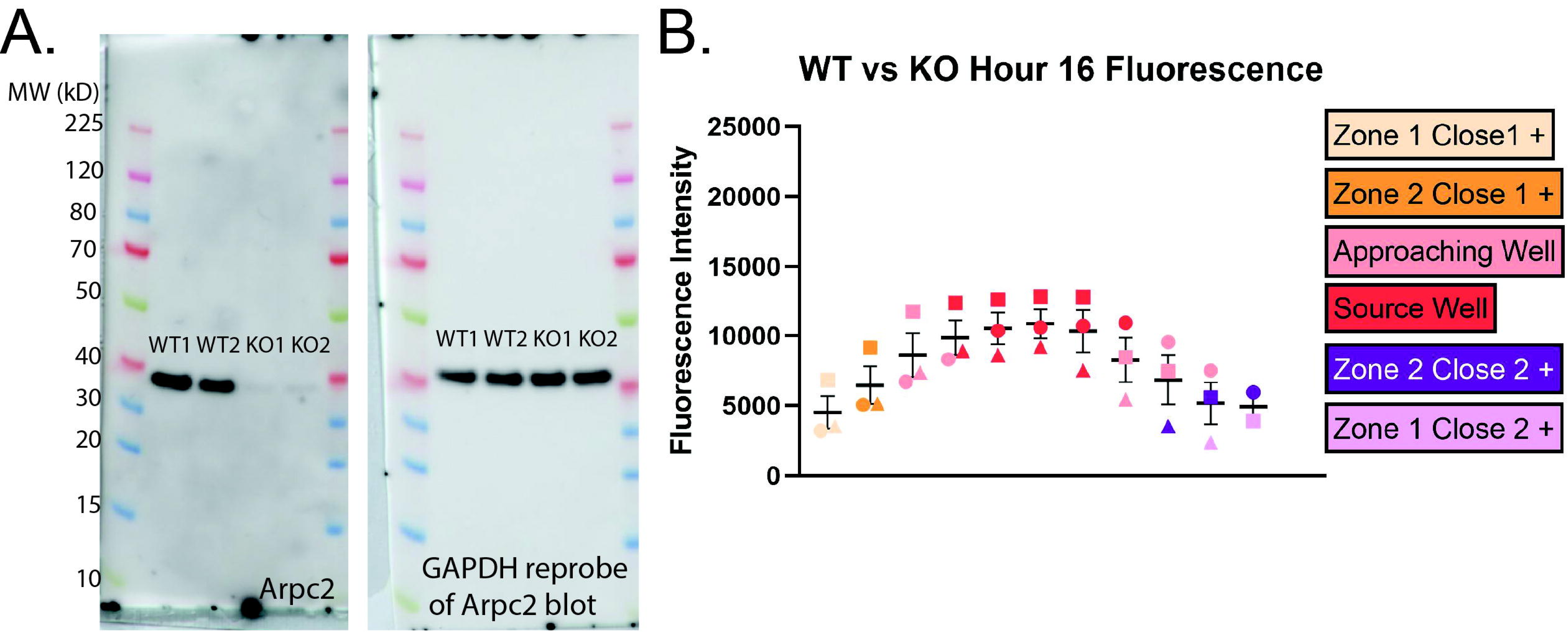

bioRxiv preprint doi: https://doi.org/10.1101/2025.05.15.654321; this version posted May 15, 2025. The copyright holder for this preprint (which was not certified by peer review) is the author/funder. This article is a US Government work. It is not subject to copyright under 17 USC 105 and is also made available for use under a CC0 license.

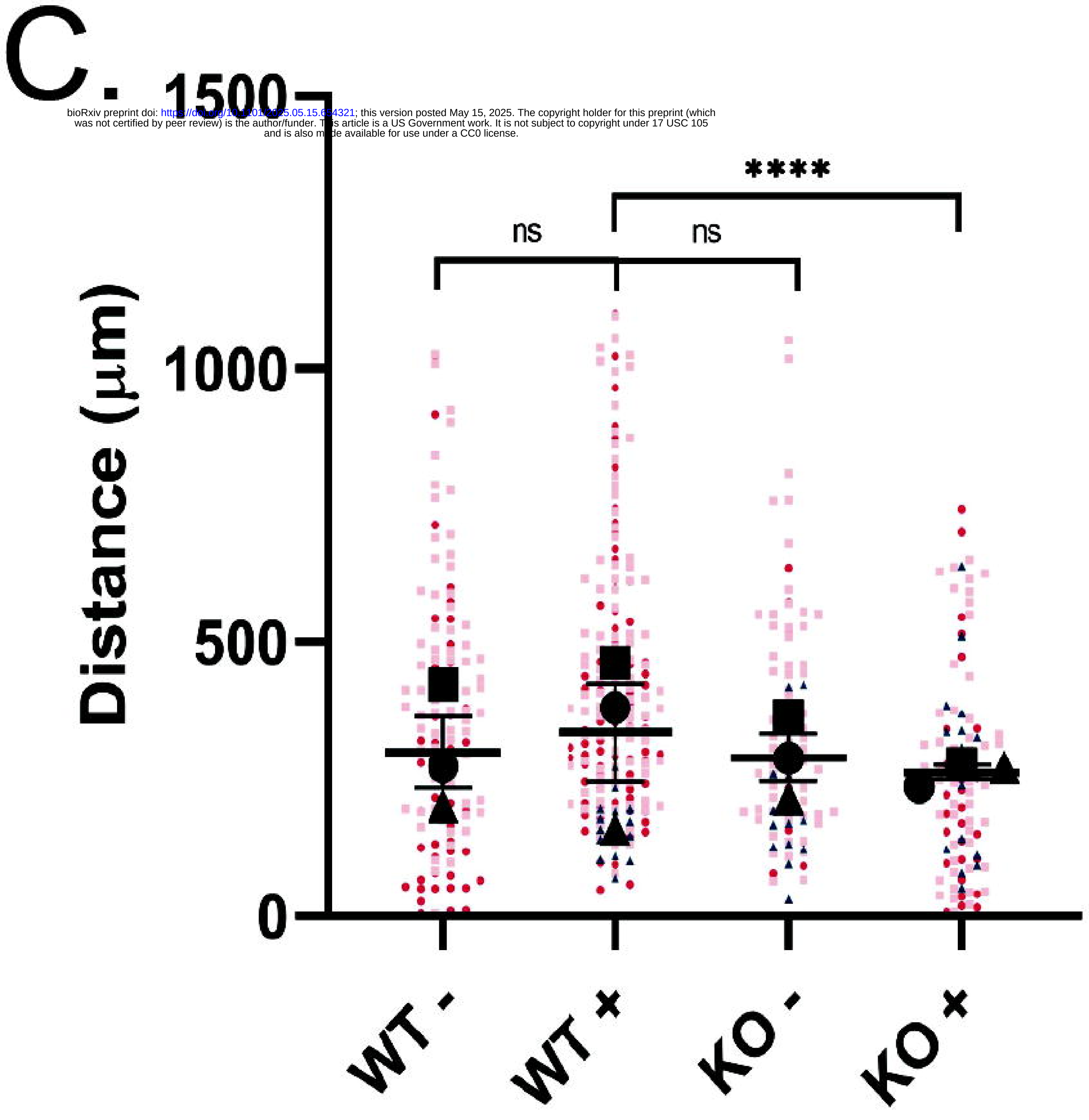

Stinson et al., Supplemental Figure 5

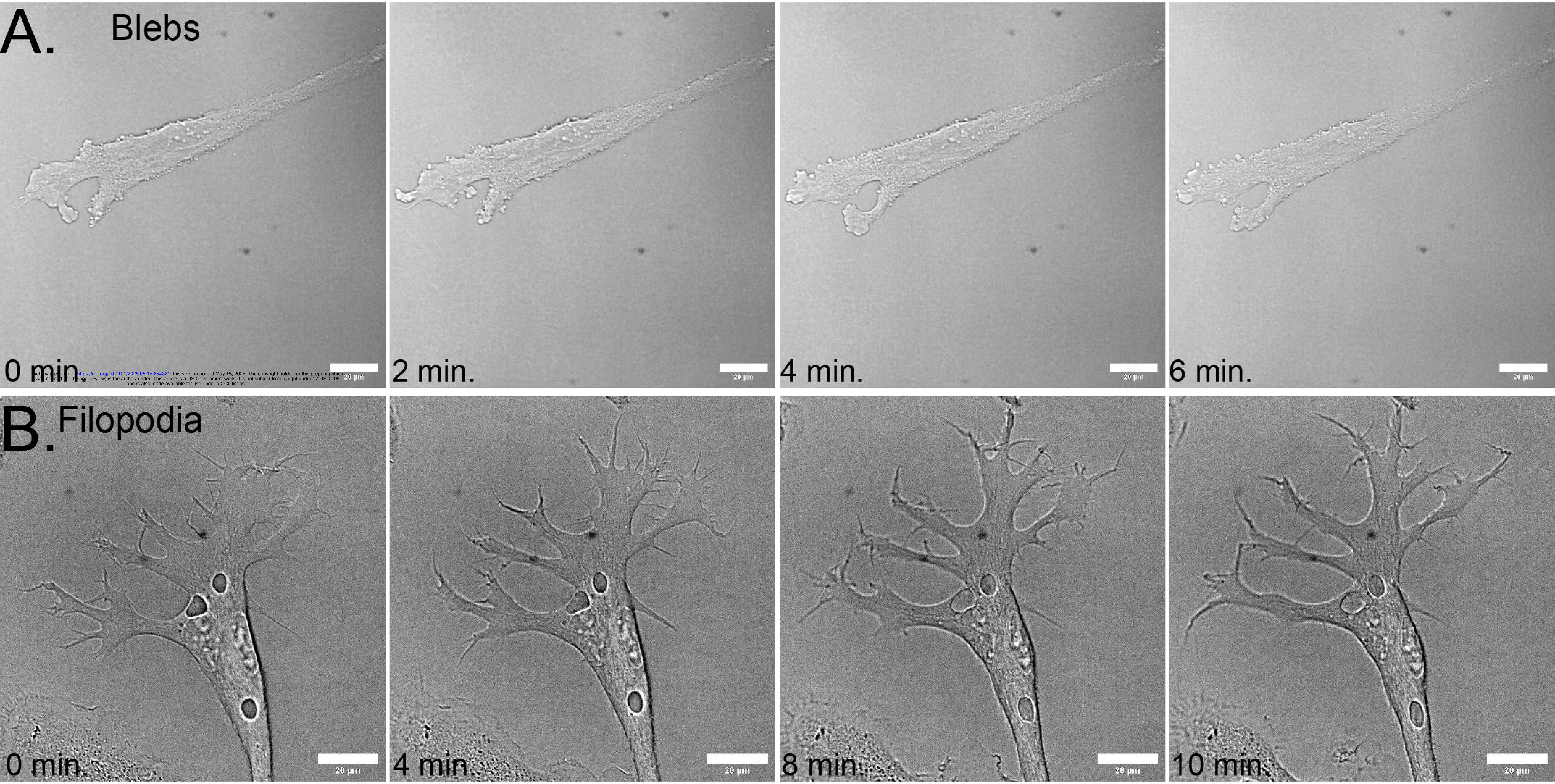

bioRxiv preprint doi: https://doi.org/10.1101/2025.05.15.654321; this version posted May 15, 2025. The copyright holder for this preprint (which was not certified by peer review) is the author/funder. This article is a US Government work. It is not subject to copyright under 17 USC 105 and is also made available for use under a CC0 license.

Stinson et al., Supplemental Figure 6
